# Protocol for sterile tumor cell implantation into the spleen or cecal wall of germ-free mice

**DOI:** 10.1016/j.xpro.2026.104835

**Published:** 2026-09-15

**Authors:** Thi Trang Le, My-Lan Pianka, Christoph K. Stein-Thoeringer, Regula Burkhard, Lukas F. Mager

**Affiliations:** 1 M3 Research Center, Faculty of Medicine, University of Tübingen, Tübingen, Germany; 2 Department of Internal Medicine I, Faculty of Medicine, University of Tübingen, Tübingen, Germany; 3 Department of Infectious Diseases, Medical Microbiology and Hygiene, Medical Faculty Heidelberg, Heidelberg University, Heidelberg, Germany; 4 University Hospital Heidelberg, Heidelberg, Germany

**Keywords:** Cancer, Microbiology, Model Organisms

## Abstract

Establishing tumor models in germ-free (GF) mice is essential to study the effects of microbiome on cancer progression and treatment responses. Here, we present a protocol for the intrasplenic and orthotopic cecal wall implantation of colorectal cancer cells in GF mice under sterile conditions. Intrasplenic injection models late-stages of liver metastasis, whereas orthotopic cecal wall implantation recapitulates the complete metastatic cascade. We describe procedures for material preparation, sterile surgery, and sterility monitoring to ensure successful tumor establishment while maintaining GF status.

## Highlights

• Instructions on preparing sterile materials for germ-free surgery
• Standardized, aseptic workflow to arrange a sterile workspace
• Step-by-step protocol for abdominal surgery in germ-free mice
• Guide to monitor sterility of germ-free mice post-surgery

## Abstract (editor highlights)

Publisher’s note: Undertaking any experimental protocol requires adherence to local institutional guidelines for laboratory safety and ethics.

## Graphical abstract

**Figure:**
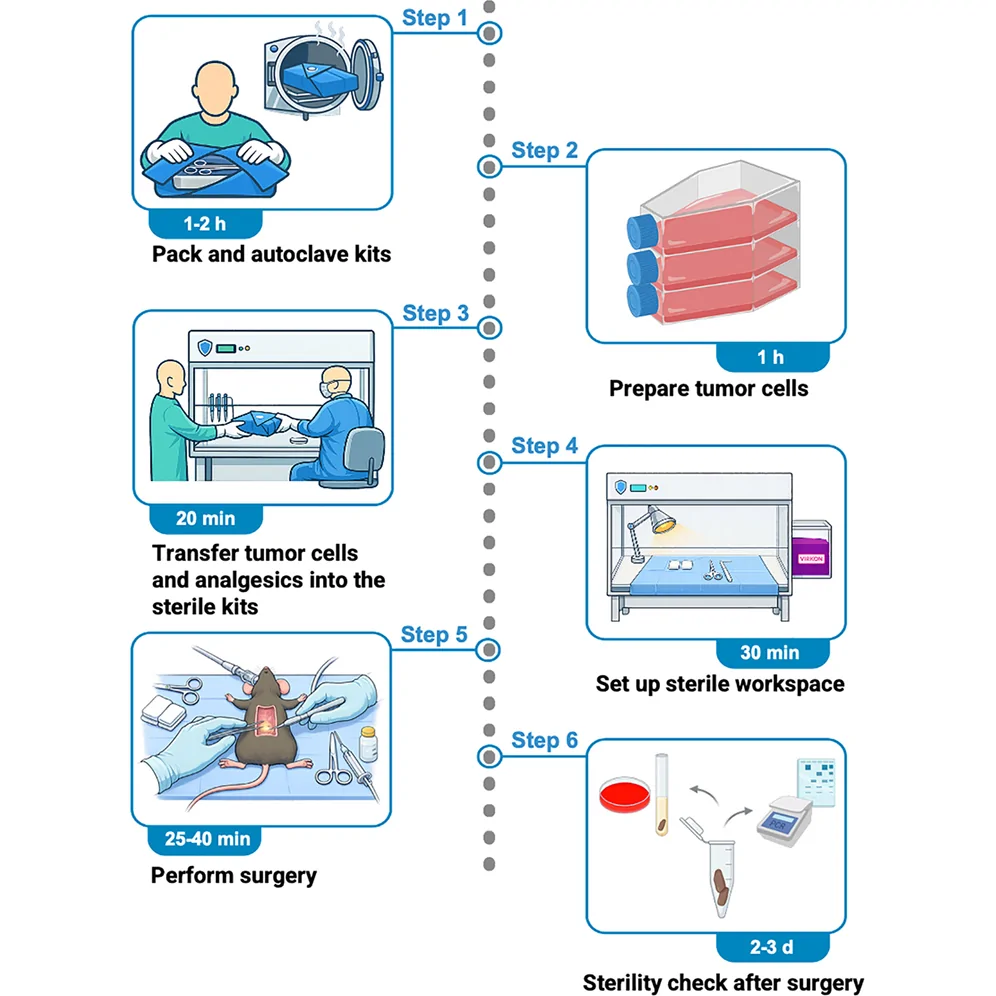

## Before you begin

The microbiome has been demonstrated to exert both supportive and suppressive effects on tumor growth, metastasis, and responses to cancer therapy.^1^^,^^2^^,^^3^ Tumor models in GF and gnotobiotic mice therefore provide powerful platforms to dissect microbe-host interactions in cancer. Depending on the research question, colorectal cancer progression can be modeled using either orthotopic cecal wall implantation or intrasplenic injection, both of which are well-established surgical approaches.^4^^,^^5^ Orthotopic cecal wall implantation has been applied in both syngeneic and xenograft colorectal cancer models.^6^ It allows investigation of primary tumor growth together with the complete metastatic cascade, including local invasion, intravasation, dissemination, and spontaneous metastatic colonization. Intrasplenic injection, in contrast, delivers tumor cells directly into the portal circulation, providing a reproducible model of hepatic colonization and experimental liver metastasis while bypassing the early steps of the metastatic cascade.^4^

The protocol described below adapts both surgical approaches for use in GF mice and provides a coordinated two-person sterile workflow within a biosafety cabinet (BSC). In this study, MC38, a murine colon adenocarcinoma cell line, was used; however, the workflow can be used for any other tumor cell lines requiring abdominal implantation in GF mice.

We used inhalation anesthesia with isoflurane and perioperative analgesia with carprofen and lidocaine. This regimen provided stable anesthesia and fast postoperative recovery in GF mice, with no observed anesthesia-related mortality. Previous studies have reported that GF mice may respond poorly to ketamine/xylazine-based anesthesia, and dose escalation may increase mortality risk.^7^

This protocol focuses on the implantation of colorectal cancer cells into the spleen or cecal-wall of GF mice; however, it can be easily adapted for tumor cell implantation into other abdominal organs, such as liver or pancreas in GF mice.

### Innovation

Protocols for intrasplenic and cecal wall tumor implantation for conventionally housed mice have been described step-by-step before.^4^^,^^5^ However, conducting these surgical procedures under GF conditions presents several technical challenges, including limited surgical flexibility, the need to maintain sterility during extended surgical preparation, enlarged and thin-walled cecal tissue in GF mice, altered responses of GF mice to anesthetic and analgesic regimens, and preservation of tumor cell viability and sterility throughout preparation and transport. Although protocols for inducing spontaneous tumors in GF animals have also been reported^8^; to our knowledge, this is the first published step-by-step protocol for surgical implantation of tumor cells into abdominal organs of GF mice, achieving primary tumor establishment at the intended anatomical site in all implanted animals (5/5 intrasplenic- and 5/5 orthotopic cecal wall implantation) and liver metastasis in most animals (5/5 intrasplenic- and 4/5 orthotopic cecal wall implantation) while preserving axenic conditions.

### Institutional permissions

All animal experiments were conducted in accordance with federal guidelines and were approved by the “Regierungspräsidium” Tübingen, Germany (protocol numbers: M 06/22 G and M 26/24 G).

### Mice

Germ-free C57BL/6J mice were bred and maintained in sterile flexible film isolators (NKP) at the M3 Germfree facility (M3GF), M3 Research Center, University of Tübingen. All mice had access to food and water *ad libitum* and were maintained at an ambient temperature of ∼24°C, humidity of ∼45%, and with 12-h light-dark cycle. GF status was routinely confirmed by a combination of aerobic and anaerobic cultures, SYTOX green DNA staining and 16S rRNA PCR of fecal pellets. Male and female mice between 7–12 weeks were used in this study. During the experiments, GF mice were housed in HEPA filtered isocages (Tecniplast). The GF status of animals was confirmed during the experiment using culture-dependent (aerobic and anaerobic incubation in BHI medium) and -independent (16S rRNA PCR) methods. Fecal pellets collected before and after surgery were analyzed as described in section “sterility monitoring after surgery”.

### Preparation for surgical supplies

**Timing: 2 days, for preparation. Hands-on time 2 h. It is recommended that all preparations are completed at least 1 day before surgery**

***Note:*** In this protocol, all surgical materials and supplies are double-wrapped in autoclavable drapes prior to autoclaving. The inner drape is wrapped using the rectangular fold technique, while the outer drape is wrapped using the diamond fold technique. A detailed step-by-step description of the double-wrapping procedure for autoclaving is described elsewhere.^9^

**Figure 1:**
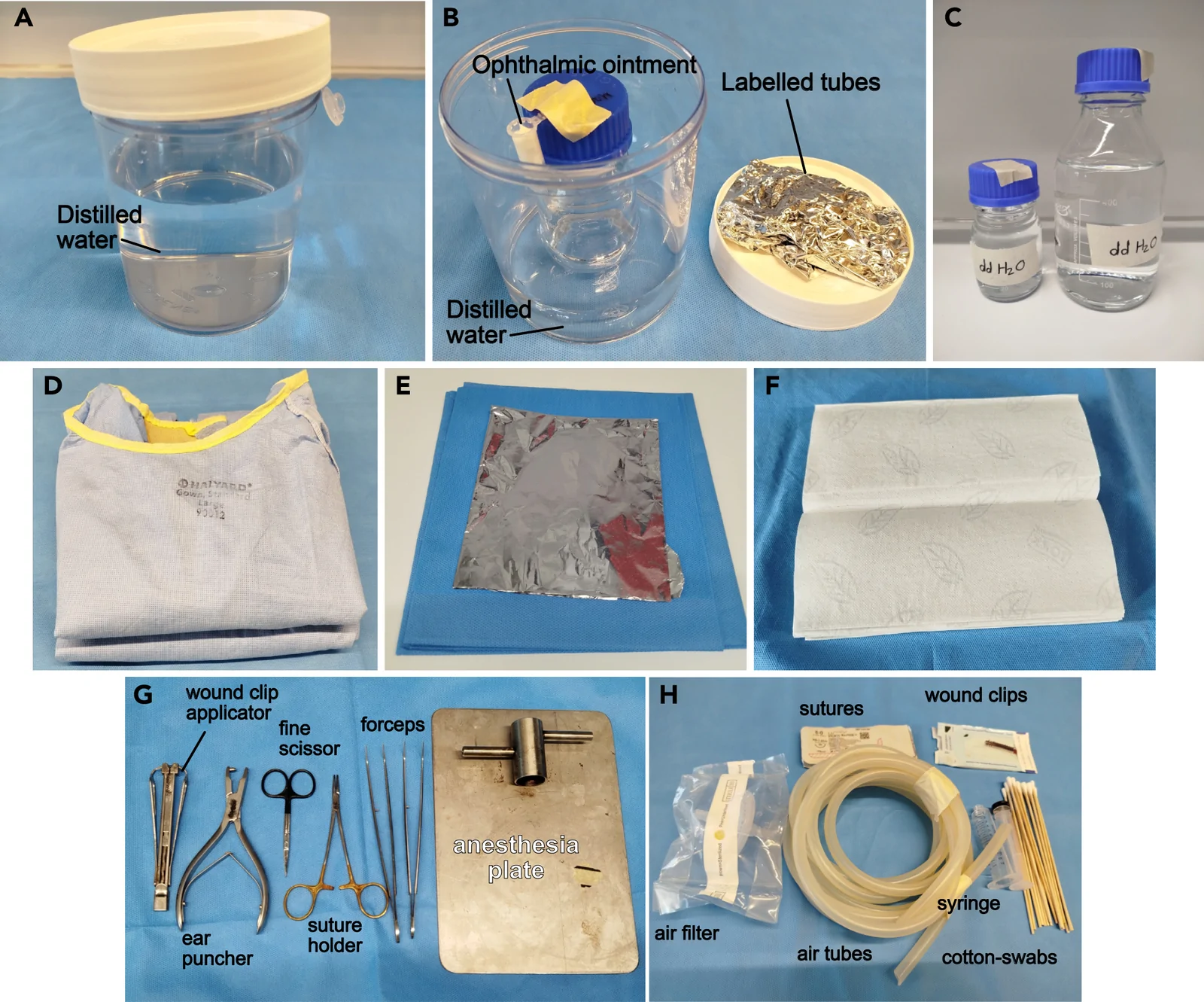
Preparation of supplies for sterilization prior to GF surgical procedures (A) Plastic container filled with distilled water for preparation of sterile ice. (B) Preparation of the cell/reagent kit consisting of a glass bottle placed in distilled water, ophthalmic ointment attached to the bottle cap, and labelled microcentrifuge tubes wrapped in aluminum foil. (C) Autoclaved distilled water stored in glass bottles before use. (D–G) Surgical gown, (E) Surgical drapes and aluminum foil, (F) Tissue paper, (G) Baked metal surgical instruments and accessories (scissors, forceps, suture holder, wound clip applicator, ear puncher, and anesthesia plate) prepared for double-wrapping in drapes and autoclaving. (H) Air filter, air-connecting tubes, sutures, wound clips, cotton swabs, and syringes.
  d. Autoclave using the liquid sterilization cycle at 121°C for 30 min with slow exhaust.
  e. Allow it to cool down to 20°C–22°C.
  f. Store at −20°C for at least 24 h to ensure complete freezing.
2. Prepare sterile cell/reagent kit for transporting cells and other reagents to the GF facility.
  a. Add 200 mL of distilled water into a 1 L plastic container.
  b. Place a 100 mL glass bottle inside the container.
  c. Transfer ophthalmic ointment into a 2 mL microcentrifuge tube.
  d. Attach the opened ophthalmic ointment tube to the glass bottle while keeping the lid of the glass bottle loose (Figure 1B).
  e. Label microcentrifuge tubes for cells, and additional reagents (e.g., lidocaine and carprofen).
  f. Wrap the labelled tubes in aluminum foil and place them on top of the container’s lid to prevent contact with the water inside the container (Figure 1B).
    **CRITICAL:** Carefully secure the ophthalmic ointment tube to the glass bottle with autoclave tape to prevent the tube from dropping down during the autoclaving process.
  g. Place an Eppendorf tube between the lid and the container body so that steam can enter the container during the autoclave cycle.
  h. Close the container and double-wrap the entire kit.
  i. Autoclave using the liquid sterilization cycle at 121°C for 30 min with slow exhaust.
  j. Allow the kit to cool down to 20°C–22°C.
  k. Store at −20°C for at least 24 h to ensure complete freezing.
3. Prepare sterilized distilled water for surgery.
  a. Autoclave distilled water in glass bottles and allow them to cool to 20°C–22°C before use (Figure 1C).

***Note:*** Prepare around 20 mL of distilled water per mouse for post-injection rinsing to remove residual tumor cells from tissue surfaces and the abdominal cavity.

1. Prepare sterilized ice for maintaining cell viability during surgery.
  a. Add approximately 800 mL of distilled water into a 1 L plastic container.
  b. Place an Eppendorf tube between the lid and the container body so that steam can enter the container during the autoclave cycle.
  c. Double-wrap the container with surgical drapes (Figure 1A).

4. Prepare surgical gown for surgeon.
  a. Double-wrap the surgical gowns with 2 layers of surgical drapes (Figure 1D).
  b. Autoclave at 121°C for 20 min.
5. Prepare surgical drapes to cover surgical area.
  a. Fold each drape in an accordion pattern along both axes to form a square approximately 25 cm × 25 cm.
  b. Place a sheet of aluminum foil on top of the folded drape (Figure 1E).
    ***Note:*** Aluminum foil measuring approximately 20 × 30 cm is sufficient to cover the handle of the dunk tank door for sterile handling.
  c. Double-wrap the drape and aluminum foil.
  d. Autoclave at 121°C for 20 min.
6. Prepare sterile tissue paper.
  a. Double-wrap tissue paper and autoclave at 121°C for 20 min (Figure 1F).
7. Prepare surgical instruments.
  a. Bake all metal instruments, including anesthesia plate, scissors, forceps, wound clip applicator/remover, and ear puncher, at 180°C for 3 h (Figure 1G).
  b. Allow the instruments to cool down to 20°C–22°C.
  c. Double-wrap the instruments in 2 layers of drapes and autoclave at 121°C for 20 min.
8. Double-wrap air-connecting tubes, 0.22 μm air filters, sutures, wound clips, cotton swabs, and 12 mL or 20 mL syringes and autoclave at 121°C for 20 min (Figure 1H).

***Note:*** Use sterilization indicator tape to confirm successful autoclaving. After sterilization, store all non-liquid containing packages prepared in steps 4 to 8 at 60°C for 12–16 h before use to ensure complete drying and minimize the risk of recontamination.

### Culturing tumor cells for implantation

**Timing: 2–3 days, depending on the cell line**

In this protocol, the MC38 cell line is used; however, any cell line appropriate for the research question can be used. Adjust the culture conditions and growth medium according to the requirements of the respective cell line.

9. Cultivate tumor cells in RPMI supplemented with 10% FBS and 1% Pen/Strep until 70%–80% confluency in tissue cell culture flasks is reached.

**CRITICAL:** Regularly check the sterility of the cell culture supernatant using aerobic and anaerobic bacterial culturing or 16S rRNA PCR.

### Preparation of tumor cell suspension and analgesics

**Timing: Approximately 1 h**

**CRITICAL:** Thaw Matrigel on ice before use. This may require up to 1–2 h.

Perform all cell handling procedures in a clean BSC to maintain sterility. Sterilize all reagents, including PBS and trypsin-EDTA by filtration through a 0.22 μm filter before use.

10. Detach adherent cells from the cell culture flask.
  a. Remove the medium by gentle aspiration.
  b. Wash cells once with pre-warmed sterile PBS to remove residual serum.
  c. Add sufficient amount of trypsin-EDTA to completely cover the cells.
  d. Incubate at 37°C until the cells have fully detached.
  e. Immediately add up to 50 mL of PBS to rapidly dilute the trypsin-EDTA.
    **Alternatives**: Serum-containing culture medium may be used to neutralize trypsin-EDTA.
  f. Transfer the cell suspension to a 50 mL falcon tube.
11. Wash cells three times with sterile PBS by centrifugation at 300 × *g* for 5 min at 4°C.
12. Resuspend the final cell pellet in 5 mL of PBS (for cells grown in a T75 flask).
13. Determine the total cell number and adjust the cell concentration according to the selected injection model.
  a. Intrasplenic injection model: for each injection, suspend 2 × 10^5^ cells in 200 μL of PBS.
    ***Note:*** In this study, 200 μL of PBS containing 2 × 10^5^ cells was injected into the spleen to achieve consistent liver metastasis. However, for inexperienced operators, this relatively large injection volume may increase the risk of cell leakage. Injection volume can be optimized according to the experimental requirements.
  b. Cecal wall injection model: for each injection, suspend 1 × 10^6^ cells in 14 μL of PBS and mix with Matrigel to obtain a final volume of each 20 μL containing 30% Matrigel and 1 × 10^6^ cells.
    ***Note:*** Prepare at least twice the required volume of cell suspension to compensate for potential material loss during handling.
14. Store the prepared cell suspension in 50 mL falcon tubes at 4°C.
15. Prepare carprofen (1 mg/mL).
  a. Dilute 200 μL of stock solution (50 mg/mL) in 9.8 mL of 0.9% saline.
  b. Sterilize the solution by filtering through a 0.22 μm filter inside the BSC.
  c. Store in a 50 mL falcon tube until further use.
16. Prepare lidocaine (0.5% w/v).
  a. Weigh 0.025 g of lidocaine.
  b. Dissolve in 5 mL of 0.9% saline.
  c. Sterilize the solution by filtering through a 0.22 μm filter inside the BSC.
  d. Store in a 50 mL falcon tube until further use.

### Transfer tumor cell suspension and analgesics into the sterile cell/reagent kit

**Timing: Approximately 20 min**

***Note:*** Perform this procedure inside a BSC using a coordinated two-person workflow involving an assistant and an experimenter. To introduce materials into the BSC, the assistant first disinfects the outer surface of the autoclaved packages or materials with 70% ethanol, then carefully opens the outer wrapping layer and presents the sterile contents to the experimenter through the opening of the BSC without touching the inner drape. Perform this process efficiently to minimize the risk of airborne contamination.

**Figure 2:**
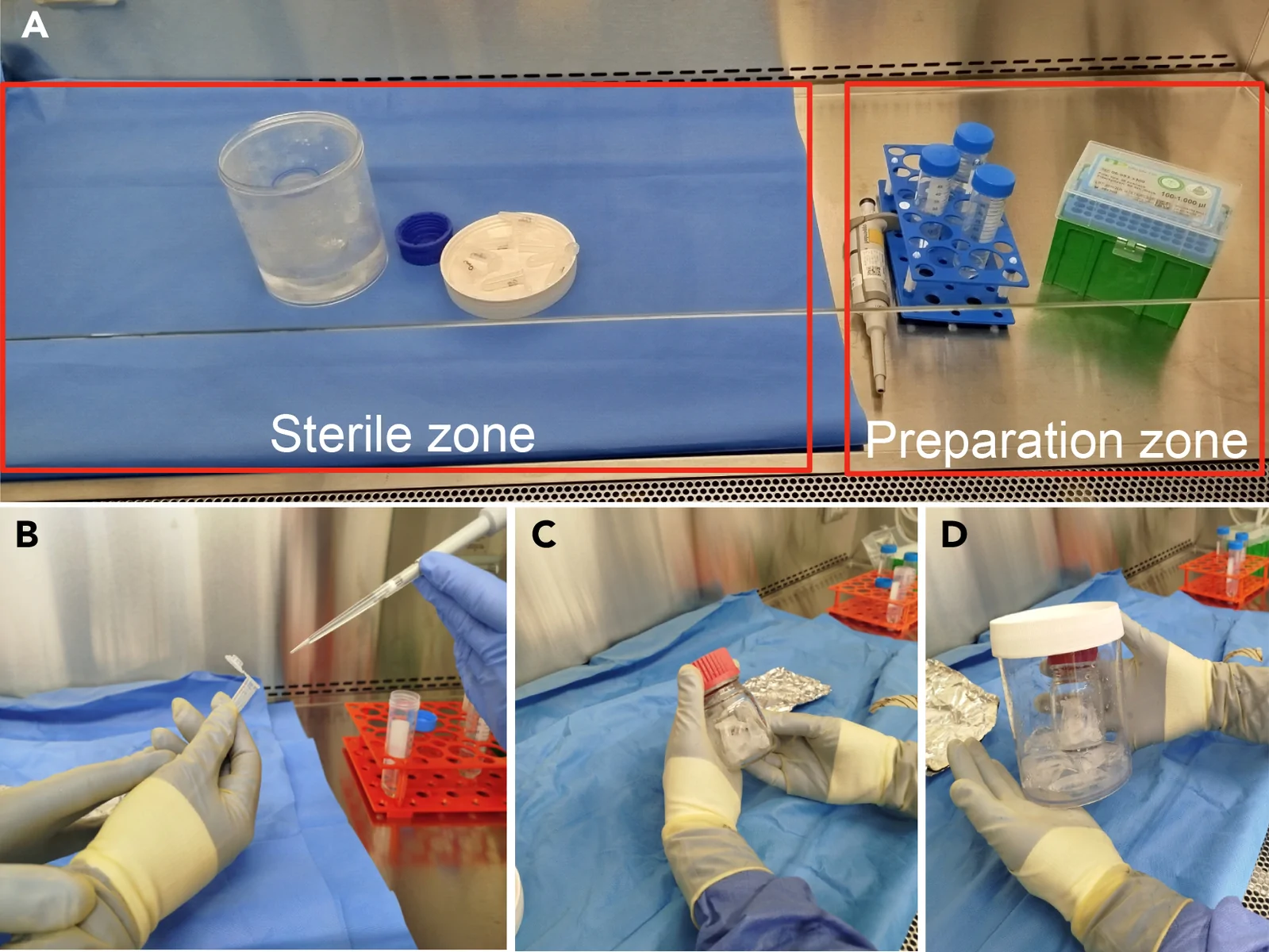
Transfer tumor cells and analgesics into the sterile cell/reagent kit (A) The BSC is divided into a preparation and a sterile working zone. (B) Two-person workflow for transferring cells and reagents into sterile cell/reagent kits. (C) Cell suspensions and reagent tubes are placed into a sterile glass bottle. (D) The sealed glass bottle containing cells and reagents is returned to the transport container filled with sterile ice.

17. Disinfect all BSC surfaces twice using 2% Virkon solution followed by two rounds of 70% ethanol.
18. Experimenter dons the sterilized gown and places both arms into the BSC (hands stay within the gown).
19. The assistant disinfects and introduces surgical gloves to the BSC for the experimenter.
20. The experimenter dons the surgical gloves without touching the external glove surfaces.
21. Establish separate sterile and preparation zones inside the BSC (Figure 2A).
  a. Cover the sterile zone with autoclaved drapes (Figure 2A).
  b. Import the sterilized cell/reagent kit and open it within the sterile area.
    ***Note:*** Freezing at −20°C may cause the glass bottle to adhere to the surrounding ice. Prior to use, allow the cell/reagent kit to equilibrate at 20°C–22°C for approximately 20 min to partially thaw the ice and facilitate removal of the bottle.
  c. Assistant disinfects all materials required for reagent transfer with 70% ethanol, including the cell suspension tubes, analgesic tubes, pipette, pipette tip box and arrange them within the preparation zone of the BSC (Figure 2A).
  d. Transfer tumor cell suspension and reagents into sterile tubes using a coordinated no-contact workflow.
    i. The experimenter holds sterile tubes within the sterile zone.
    ii. The assistant transfers cell suspension and liquid analgesics from the preparation zone without direct contact between pipette tips and sterile tubes (Figure 2B).
    iii. The experimenter places all sterile tubes containing ophthalmic ointment, tumor cells and analgesics into the glass bottle inside the container with sterile ice (Figure 2C).
    iv. The experimenter closes the lid of the container tightly (Figure 2D).
    v. Store kit at 4°C until surgery.
      **CRITICAL:** Maintain strict separation between sterile and preparation zones during the entire procedure.
      ***Note:*** Initiate surgery as soon as possible after cell preparation to maintain optimal cell quality and viability.
      **Optimal:** Assess cell viability using trypan blue staining before and after surgery to confirm that the cells remain viable throughout the entire implantation procedure.

## Key resources table

**Table:** 

REAGENT or RESOURCE	SOURCE	IDENTIFIER
**Bacterial and virus strains**		

*E. coli* BW25113	DSMZ	DSM 27469

**Chemicals, peptides, and recombinant proteins**		

Ethanol 99% (denatured/technical grade)	SAV Liquid Production GmbH	Cat#ETO-5000-99-1
Virkon S	Lanxess	Cat#1060088
Gibco™ Dulbecco’s Balanced Salt Solution	Thermo Fisher Scientific	Cat#14190094
Isotonic Saline Solution (0.9%)	Fresenius Kabi Germany	Cat#00808765
Carprofen	Zoetis	Rimadyl® injectable (50 mg/mL)
Lidocaine	Sigma-Aldrich	Cat#L7757
Isoflurane	CP-Pharma	Cat#1214
Bepanthen eye and nose ointment	Bayer Vital GmbH	Cat#01578681
RPMI 1640 Medium	Thermo Fisher Scientific	Cat#11875-093
FBS Superior	Sigma Aldrich	Cat#S0615
Penicillin-Streptomycin	Sigma Aldrich	Cat#P4333
Trypsin-EDTA solution	Sigma-Aldrich	Cat#T4174
Corning® Matrigel® Basement Membrane Matrix	Merck	Cat#CLS354234-1EA
Brain Heart Infusion Broth (BHI)	Sigma-Aldrich	Cat#53286
5× GoTaq® Flexi Reaction Buffer	Promega	Cat#M8911
dNTP Mix	Promega	Cat#U1420
GoTaq G2 Hot Start Polymerase	Promega	Cat#M7848
BenchTop 1kb DNA Ladder	Promega	Cat#G754A
BD BBL Columbia agar with 5% Sheep Blood Plate	BD	Cat#221263
Agarose Standard	Carl Roth	Cat#3810.2
SYTOX Green Nucleic Acid Stain	Invitrogen	Cat# S7020

**Critical commercial assays**		

DNeasy PowerSoil Pro Kit	QIAGEN	Cat#47016

**Experimental models: Cell lines**		

MC38	Kerafast	ENH204-FP

**Experimental models: Organisms/strains**		

Germ-Free C57BL/6J: male/female, 6-12 weeks	M3 Germ-free facility	RRID:MGI:2159769

**Oligonucleotides**		

fD1: AGA GTT TGA TCC TGG CTC AG	Integrated DNA Technologies	NA
fD2: AGA GTT TGA TCA TGG CTC AG	Integrated DNA Technologies	NA
rP1: ACG GTT ACC TTG TTA CGA CTT	Integrated DNA Technologies	NA

**Other**		

ISO-TECH60 Biosafety cabinet with dunk tank	Tecniplast	NA
ISOcage P maintain system	Tecniplast	NA
Veterinary oxygen concentrator	RWD	ROC-5A
Gas evacuation apparatus	RWD	Model R546 Pro
Vaporizer for isoflurane	RWD	Model R5835
Animal Temperature Heating Pad w/PID, Controller	Somi Science	HT-15002i
Heating lamp	Bochem Laborbedarf	NA
Gas Venting Filters, 0,22 μm	VWR	Cat#VWRI210-000126
Air connection tubes	Carl Roth	Cat#27227
Anesthesia plate	NA	NA
Surgical sterilization drapes	Medline	Cat#GEM2140-EU
Surgical gown	Halyard	Cat#90012
Biogel Super-Sensitive surgical gloves	Mölnlycke	Cat#82270
Jars Nalgene® style 2118 (1000 mL)	Thermo Fisher Scientific	Cat#Z380261
Autoclave tape	3M	Cat#1322/24
Sterile filter 0,2 μm	Sartorius	Cat#10527401
Fine Scissors - ToughCut	Fine Science Tools (F.S.T)	Cat#14058-09
London Forceps 16 cm	F.S.T	Cat#11080-02
Halsey Micro Needle Holder	F.S.T	Cat#12500-12
Reflex 7mm Wound Clip Kit (applier, wound clips, & remover)	Stoelting	Cat#59038
VICRYL RAPIDE® (polyglactin 910) Suture 0,7 m	Johnson & Johnson	Cat#V2130H
Cotton-swab	Omnilab	Cat#52737
Single-packed insulin syringe 0.5 mL	B. Braun	Cat#00465650
BD Plastipak™ 20 mL or 12 mL Luer-Lok syringe	BD	Cat#300629; 300912

## Step-by-step method details

### Setup of the surgical field inside the BSC

**Timing: 30 min**

**Figure 3:**
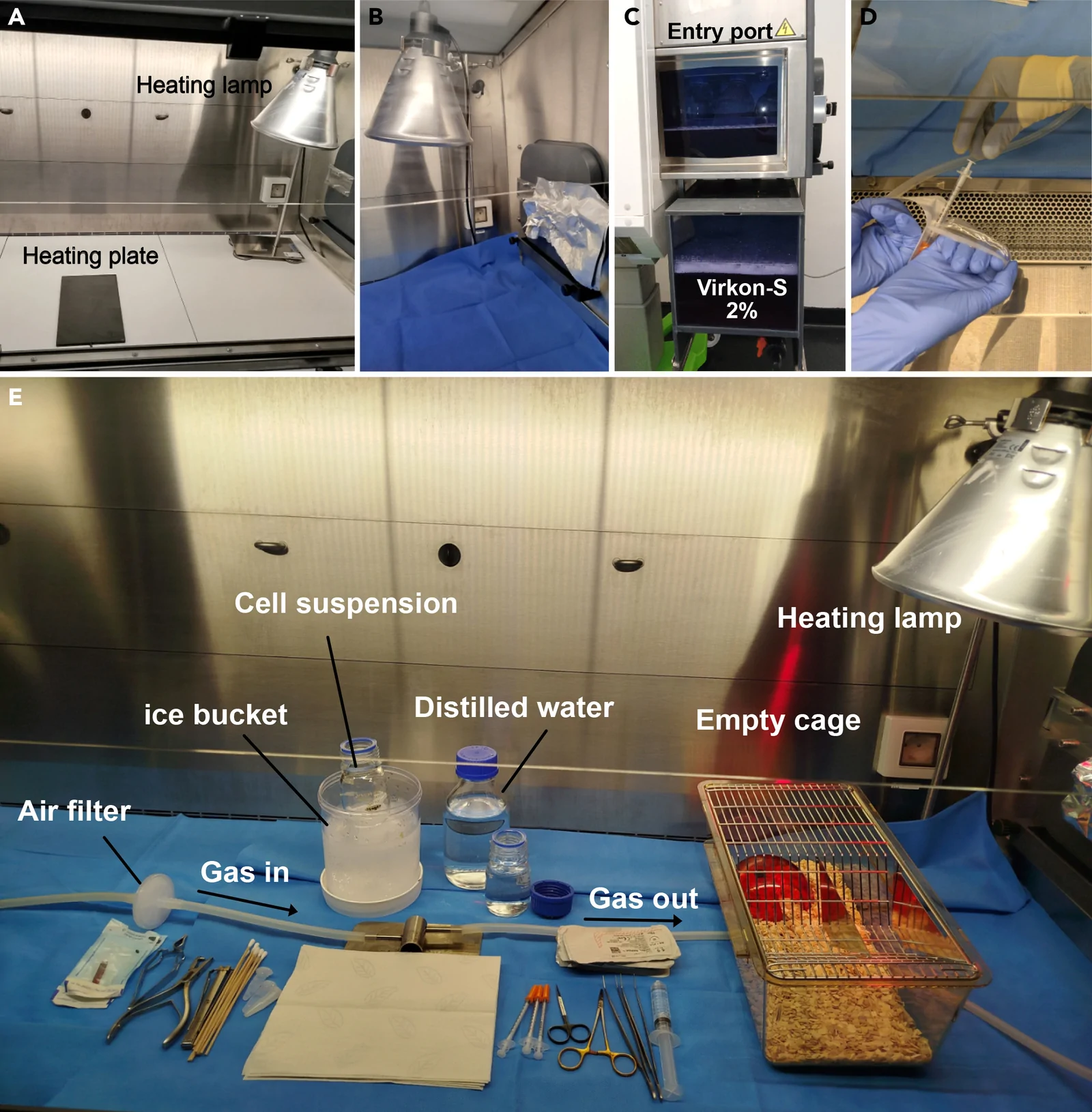
Setup of the sterile surgical field inside the BSC (A) Heating plate and heating lamp positioned inside the BSC before preparation of the sterile surgical field. (B) Working area covered with sterile drapes and the handle of the inner dunk tank door covered with aluminum foil. (C) Integrated dunk tank system with 2% Virkon-S used for sterile transfer of GF mice and surgical materials into the BSC. (D) Transfer of an insulin syringe into the BSC using a two-person coordinated workflow. (E) Fully assembled sterile surgical field inside the BSC for GF abdominal surgery, including anesthesia system, surgical instruments, cell suspension kit, heating plate, and an empty isocage.

***Note:*** To ensure GF conditions for your experiment, conduct the next steps in a specialized animal facility equipped with engineered infrastructure for housing GF mice. In this study, we utilize the BSC equipped with a dunk tank system to sterilize (Virkon-S) and import materials into the sterile BSC. The dunk tank is a disinfectant-filled transfer chamber. Materials are placed into the chamber, immersed in Virkon-S 2% for surface disinfection, and then retrieved from the sterile side of the BSC without compromising sterility (Figure 3C).

This procedure requires a coordinated two-person workflow consisting of a surgeon and an assistant.

1. Disinfect all BSC surfaces twice using 2% Virkon-S solution followed by two rounds of 70% ethanol.
2. Disinfect the heating plate and heating lamp twice with 70% ethanol and place them inside the BSC. Then disinfect both again with 70% ethanol (Figure 3A).
3. Set the temperature of the heating plate to 41°C.

***Note:*** The heating plate will be covered with sterile drapes, anesthesia plate, and tissue paper during surgery. The effective surface temperature reaching the mice is approximately 30°C. It is recommended to validate the temperature of your own setup before surgery.

4. Perform sterile gowning and gloving of the surgeon, as described in step 18–20 of the section before you begin.

***Note:*** To introduce materials into the BSC, the assistant first disinfects the outer surface of the autoclaved packages or materials with 70% ethanol, then carefully opens the outer wrapping layer and presents the sterile contents to the surgeon through the opening of the BSC without touching the inner drape. Perform this process efficiently to minimize the risk of airborne contamination.

5. Import sterile drapes with aluminum into the BSC and cover the entire working area with the sterile drapes.
6. Cover the handle of the inner dunk tank door with sterile aluminum foil (Figure 3B).
7. Import distilled water bottles through the dunk tank system (Figure 3C).
8. Import single packed insulin syringes into the BSC (Figure 3D).
9. Transfer all surgical supplies into the BSC and arrange the surgical field as shown in Figure 3E.
10. Assemble the anesthesia system.
  a. Position the anesthesia plate on the sterile surgical field directly above the heating plate.
  b. Connect one air tube between the anesthesia plate and the waste gas collection container outside the BSC.
  c. Connect a short air tube between the anesthesia plate and a 0.22 μm air filter before routing the tube outside the BSC.
  d. Connect the external end of the tube from step 10c to the gas supply (Oxygen and Isoflurane mixture) outside the cabinet.
11. Transfer the container filled with sterile ice into the BSC.
12. Transfer the glass bottle containing tumor cell suspension and reagents into the BSC.

**CRITICAL:** Place the cells immediately on sterile ice to maintain cell viability.

***Note:*** For consecutive surgeries on mice within the same gnotobiotic (GF or colonized) experimental group, the same sterile surgical instrument set may be used, as all animals share the same microbial status. A new sterile syringe is used for each mouse to ensure consistent injection performance. Before performing surgeries on mice from a different experimental group or with a different microbial status, all surgical instruments and consumables are replaced with a freshly sterilized set to prevent cross-contamination between groups.

### Surgical procedure

**Timing: 25–40 min/mouse including time for analgesics absorption and recovery time**

Of note, detailed protocols for intrasplenic and cecal wall injection of tumor cells in conventionally housed mice have been described step-by-step before.^4^^,^^5^ Here, we describe adapted protocols designed to accommodate GF working conditions and the unique physiological characteristics of GF mice.

In this protocol, isoflurane anesthesia was selected as it allows rapid adjustment of anesthetic depth during prolonged sterile procedures and may provide improved tolerability in GF mice. Carprofen (1 mg/mL) was administered subcutaneously at a dose of 10 mg/kg into the neck fold, with the injection volume adjusted according to the individual body weight of each mouse. Lidocaine (0.5%, 5 mg/mL) was administered subcutaneously around the prospective abdominal incision site at a volume of 50 μL per mouse. Alternative analgesic regimens may be used in accordance with approved animal protocols and institutional guidelines.

13. Transfer the animal into the BSC through the dunk tank containing 2% Virkon-S (Figure 4A).

**Figure 4:**
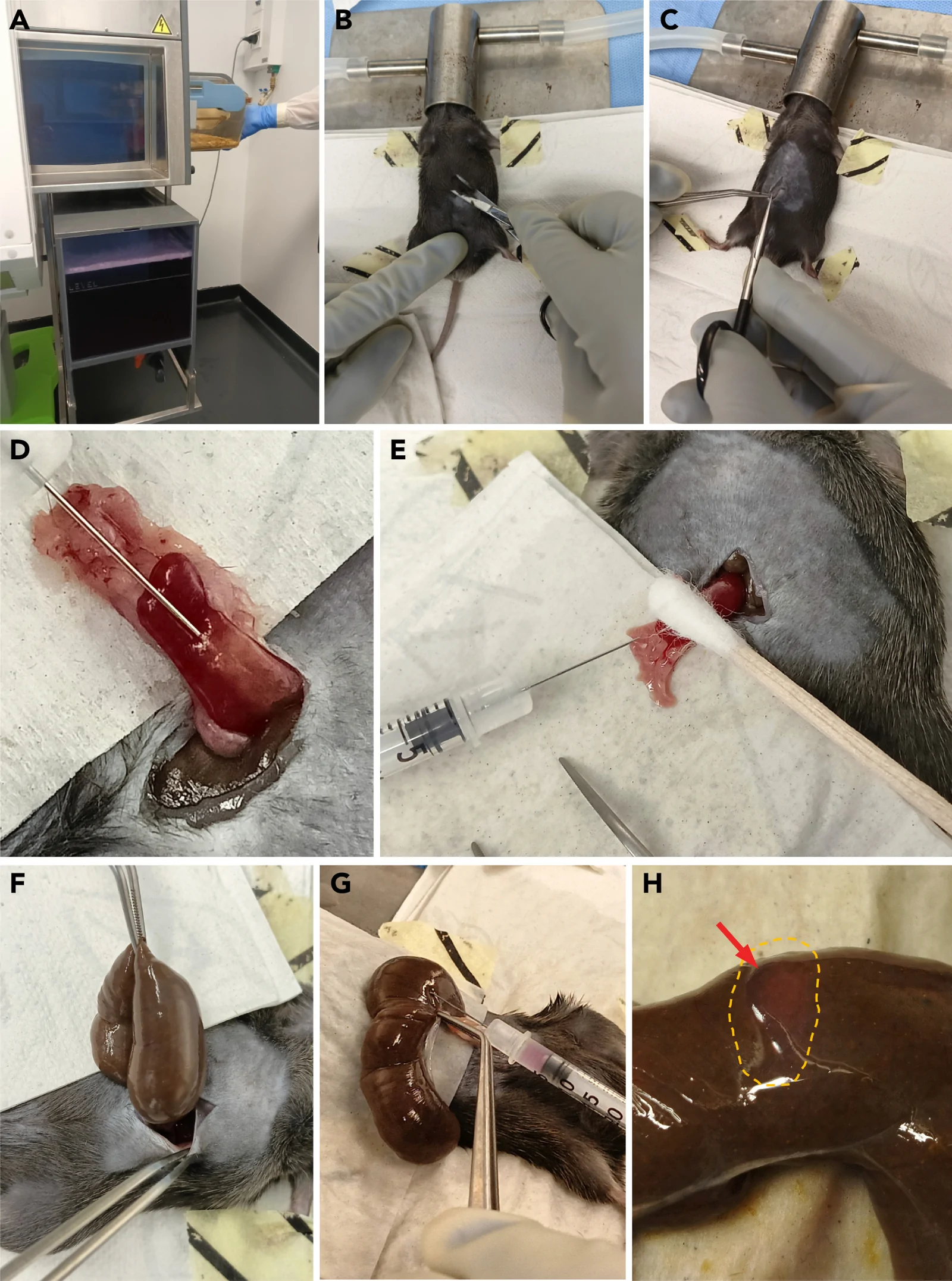
Preparation of GF mice and representative surgical procedures inside the BSC (A) Import GF mice via the dunk tank system. (B) Preparation of a GF mouse for abdominal surgery, including hair removal with the scissor. (C) Midline abdominal incision to expose the spleen and cecum. (D) Intrasplenic injection of tumor cells. (E) A cotton swab is applied to absorb leaking cells after injection. (F) Mobilization and positioning of the cecum for tumor cells implantation. (G) Syringe positioning for injection into the cecal wall. (H) A representative successful cecal wall injection showing a distinct bleb on the cecal surface.
14. Administer pre-operative analgesia (carprofen, 10mg/kg) and return the animal to its home cage for approximately 20 min to allow analgesic absorption before anesthesia induction.
15. Place the animal on the pre-warmed anesthesia plate with the nose positioned inside the anesthesia mask.
16. Induce anesthesia using 5% isoflurane and maintain anesthesia at 1.7% isoflurane with an airflow rate of 1.5–2 L/min throughout the procedure.

***Optional:*** Induce anesthesia with 5% isoflurane in a chamber until the mouse is fully anesthetized, then transfer the animal to the anesthesia plate and maintain at 1.7% isoflurane via nose mask.

17. Apply ophthalmic ointment to prevent corneal drying during anesthesia.
18. Secure the animal in a supine position and prepare the abdominal surgical site according to the selected implantation procedure.
19. Carefully remove hair from the abdominal area using scissors (Figure 4B).
20. Inject 50 μL of 0.5% lidocaine subcutaneously surrounding the incision site and allow approximately 5 min for local analgesic absorption.
21. Lift the skin up and perform a midline abdominal incision (Figure 4C).
22. Gently separate the skin and the underlying abdominal wall.
23. Make an incision along the linea alba to access the abdominal cavity.
24. Perform tumor cell implantation into the spleen or cecal wall as described below:
  a. Intrasplenic injection.
    i. Carefully mobilize the spleen onto a sterile moistened tissue paper or surgical gauze.
    ii. Inject up to 200 μL of tumor cell suspension into the central splenic parenchyma using an insulin syringe (0.30G, 12 mm). Maintain the needle in place for 5–10 s after injection to minimize leakage (Figure 4D).
    iii. Slowly withdraw the syringe and apply gentle pressure with a sterile cotton swab to avoid cell leakage (Figure 4E).
      **Optimal:** Rinse the spleen with sterile distilled water.
    iv. Return the spleen to its position in the abdominal cavity.
  b. Cecal wall injection.
    **CRITICAL:** The cecum of GF mice is frequently enlarged and more fragile than in conventionally housed mice. Therefore, a sufficiently large abdominal incision should be made to facilitate gentle mobilization of the cecum and minimize the risk of mechanical damage.
    i. Carefully mobilize the cecal wall onto a sterile, moistened tissue paper or surgical gauze (Figure 4F).
    ii. Gently stabilize and flatten the cecum by using a cotton swab.
    iii. Inject the tumor cell suspension mixed with Matrigel into the sub-serosal layer of the cecal wall (depots of 20 μl, Figure 4G).
      ***Note:*** Placement of the needle into the sub-serosal layer can be technically challenging, particularly for inexperienced operators, when performing surgery at a distance inside the BSC. Surgical magnification glasses may be used to improve visualization and injection accuracy. Successful injection is indicated by the formation of a visible bleb at the injection site (Figure 4H).
    iv. Slowly withdraw the syringe.
      **Optimal:** Rinse the cecum with sterile distilled water to remove potentially leaked tumor cells from the tissue surface.
    v. Return the cecum to its position in the abdominal cavity.
25. Close the peritoneum and skin using sutures and wound clips, respectively.
26. Place the animal into an empty cage under a heating lamp and monitor post-operative recovery. Once fully recovered, return the animal back to its home cage.

### Sterility monitoring after surgery

**Timing: Variable depending on the method**

**Timing: 2–3 days for step 27**

**Timing: approximately 4 h for step 28**

In this protocol, GF status was monitored by aerobic and anaerobic cultures and 16S rRNA PCR analysis of fecal samples as outlined below. However, alternative microbiological monitoring approaches may also be suitable depending on facility-specific standards and experimental requirements.^10^^,^^11^

27. Culture-based sterility testing.
  a. Collect fecal samples from mice 2–3 days before and at least 1 week after surgery.
  b. Incubate fecal samples in appropriate broth medium (e.g., BHI medium) under aerobic and anaerobic conditions for 24 h.
  c. Plate broth cultures onto agar plates (e.g., Columbia blood agar or Schaedler agar) and incubate for an additional 24 h.
    **CRITICAL:** For anaerobic culture, the broth medium and agar plates should be placed inside the anaerobic chamber at least 24 h in advance to reduce oxygen levels in the media.
28. 16S rRNA PCR-based sterility testing.
  a. Isolate total DNA from fecal samples using the DNeasy PowerSoil Pro Kit, according to the manufacturer’s instructions.
    ***Note:*** Include *E.coli* or other bacteria culture as a positive control.
  b. Measure DNA concentration using a NanoDrop spectrophotometer.
    ***Note:*** GF fecal samples typically yield extremely low or undetectable DNA concentrations; any elevated DNA may provide an early indication of microbial contamination.
  c. Perform PCR amplification of the bacterial 16S rRNA gene using the universal bacterial primers fD1, fD2 and rP1 (final concentration: 200 nM, for primers’ sequences, please refer to the key resources table) in a 25 μL reaction containing 1x Green GoTaq Flexi buffer, dNTP Mix (200 μM), GoTaq G2 Hot Start Polymerase (0.05 units/μL) and DNA templates. Thermal cycling consisted of an initial denaturation at 94°C for 1 min, followed by 25–35 cycles of 94°C for 1 min, 43°C for 1 min, and 72°C for 2 min, with a final extension at 72°C for 7 min.^9^
  d. Run the PCR products on a 1% agarose gel at 120 V until adequate separation is achieved.

## Expected outcomes

**Figure 5:**
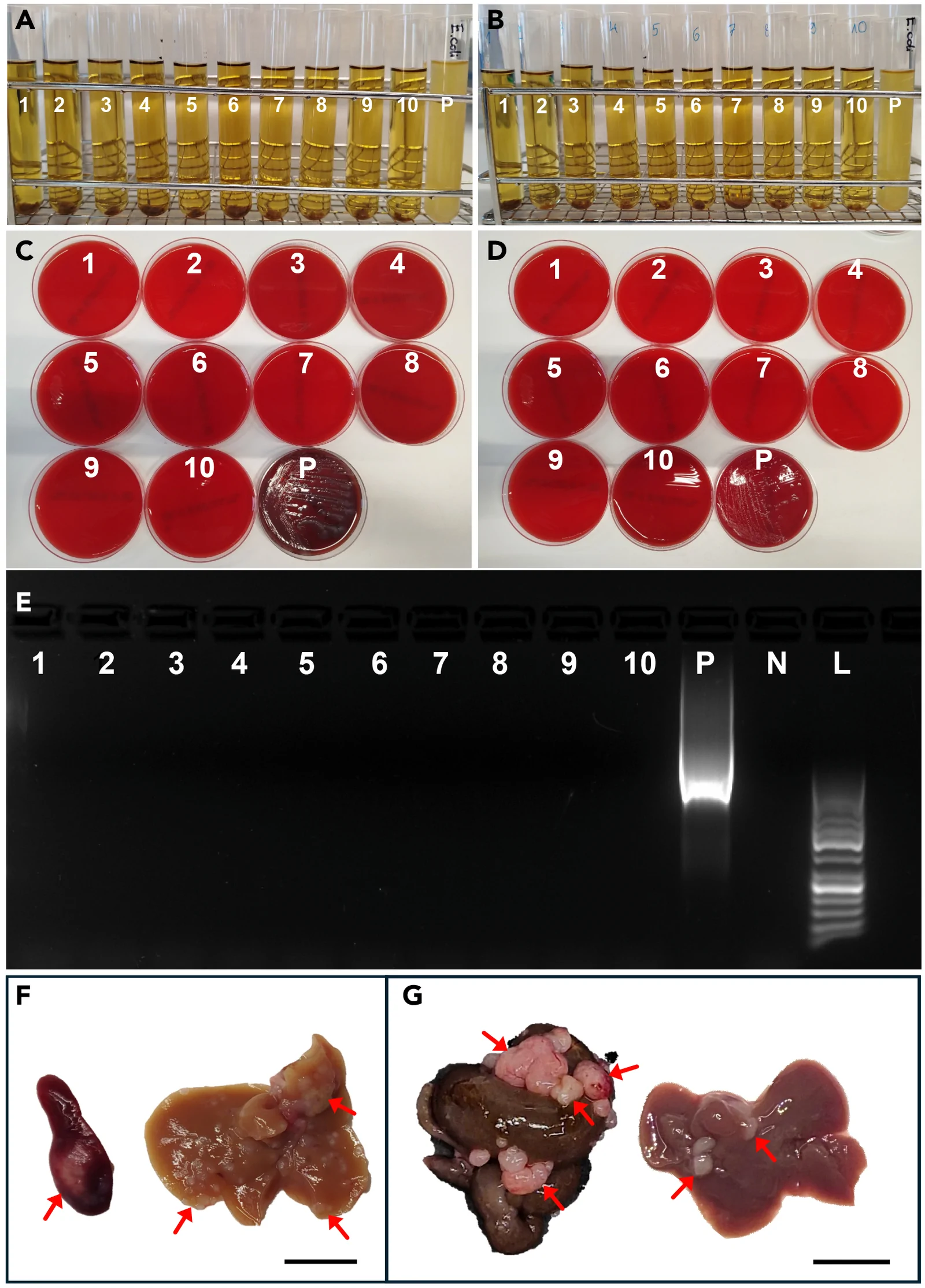
Sterility assessment and tumor growth following abdominal surgery in GF mice (A and B) Aerobic and (B) anaerobic broth cultures prepared from fecal samples collected after surgery from GF mice. (C and D) Corresponding aerobic (C) and anaerobic (D) cultures on columbia blood agar plates showing no bacterial growth in samples from GF mice, whereas positive controls demonstrated robust bacterial growth. (E) Representative 16S rRNA PCR analysis of fecal DNA samples collected 4 weeks after surgery. 1–10, fecal samples from GF mice; P, *E. coli* was used as positive control; N, negative control; L, DNA ladder. (F and G) Representative images of successful tumor implantation in spleen (F) and cecum (G) together with their corresponding livers showing metastatic lesions, 4 weeks after surgery; tumors are indicated by red arrows; scale bar, 1 cm.

Following the described surgical procedures, mice are expected to maintain GF status throughout the experiment. Fecal samples collected one-week post-surgery should show no detectable bacterial growth under aerobic or anaerobic culture conditions and no colony formation on agar plates. Consistently, 16S rRNA PCR analysis should yield negative results, indicating absence of detectable microbial contamination. In our experiments, all 10 GF mice remained free of detectable contamination for up to 4 weeks after surgery (Figures 5A–5E). Tumor formation was consistently detectable within 4 weeks after cell implantation, with localized tumor growth observed in the spleen or cecum and corresponding liver metastasis (Figures 5F and 5G). Using the colorectal cancer cell line described in this protocol, liver metastases were observed in 5/5 mice following intrasplenic injection and in 4/5 mice following orthotopic cecal wall implantation. No unintended tumor growth within the abdominal cavity was observed, indicating successful and localized delivery of tumor cells.

## Limitations

This protocol provides a reproducible method for performing tumor cell injections into the spleen and cecal wall of GF mice. However, several limitations should be considered. All surgical procedures must be performed under strictly sterile conditions within a GF environment. Although the workflow involves coordinated interaction between a surgeon and an assistant, all sterile surgical manipulations inside the BSC are performed independently by the surgeon without direct operative assistance. The extensive sterile preparation and handling requirements increase procedural complexity and may prolong surgical time especially for surgeons that are inexperienced for this specialized setup, which thereby can limit experimental throughput. In addition, performing surgeries across multiple experimental days is not recommended if identical tumor implantation conditions are required. Variations in cell preparation, handling time, storage duration, or cell viability may influence tumor establishment and contribute to experimental variability.

## Troubleshooting

### Problem 1

Loss of sterility during preparation or surgery.

Breaches in the sterile workflow may occur during transfer of materials, glove handling, or accidental contact between sterile and non-sterile surfaces inside the BSC.

### Potential solution

Maintain strict separation between sterile and preparation zones throughout the procedure. Prepare additional sterile backup packages containing gowns, drapes, surgical instruments, and consumables in case sterility is compromised during setup or surgery.

### Problem 2

Suture detachment after autoclaving (step 7).

Autoclaving may weaken attachment of sutures to surgical needles, resulting in needle detachment during wound closure.

### Potential solution

Prepare additional sterile backup sutures before surgery and verify suture integrity prior to use.

### Problem 3

Reduced tumor cell viability during cell preparation, transfer into GF facility prolonged surgical preparation (step 10–14, 21d, 24).

Extended preparation times, repeated handling of tumor cell suspensions, or prolonged storage of loaded syringes outside the cooled transport system may reduce tumor cell viability before implantation.

### Potential solution

Maintain tumor cell suspensions in sterile microcentrifuge tubes stored on autoclaved ice throughout the procedure. Return tubes to the cooled transport container immediately after handling and minimize warming by prolonged hand contact. Load syringes for each individual mouse before implantation and avoid prolonged storage of loaded syringes outside the cooled environment. Minimize repeated aspiration and dispensing of the cell suspension to reduce mechanical stress on cells. Verify cell viability at the end of the experiment.

### Problem 4

Absence of signs indicating successful tumor cell implantation (step 24).

Incorrect needle placement, insertion of the needle too deeply through the tissue, or excessive injection pressure may prevent successful deposition of the tumor cell suspension. During orthotopic cecal wall implantation, successful injection is indicated by the formation of a localized sub-serosal bleb. During intrasplenic injection, successful tumor cell deposition is indicated by transient distension of the splenic parenchyma without visible leakage.

### Potential solution

During orthotopic cecal wall implantation, insert the needle almost parallel to the cecal wall so that the needle shaft remains visible through the thin tissue and only the bevel enters the sub-serosal layer. Inject the tumor cell suspension slowly while observing the formation of a localized sub-serosal bleb. If no bleb forms after injection of a small volume, do not continue injecting the remaining cell suspension, as this may increase the risk of tissue disruption and tumor cell leakage. Instead, withdraw the needle and repeat the injection at a different site. During intrasplenic injection, gently stabilize the spleen by applying light traction to the splenic fat pad rather than grasping the splenic parenchyma directly. This minimizes compression of the spleen, reduces movement during needle insertion, and facilitates accurate needle placement while decreasing the risk of tumor cell leakage. Insert the needle into the central splenic parenchyma at a shallow angle (approximate 30° angle), ensuring that the needle tip is completely embedded within the splenic tissue without penetrating the opposite side.

### Problem 5

Cell leakage during tumor cell injection resulting in intraperitoneal tumor growth (step 24).

Incomplete sub-serosal or intrasplenic injection, excessive injection pressure, or large injection volumes may result in leakage of tumor cells from the injection site. These leaked cells can seed the peritoneal cavity, leading to unintended intraperitoneal tumor growth.

### Potential solution

To minimize the risk of tumor cell leakage, ensure correct needle placement before injection and inject the cell suspension slowly without excessive pressure. For inexperienced surgeons performing the intrasplenic injection model, a smaller injection volume (e.g., 50 μL) may reduce the risk of leakage. After completing the injection, leave the needle in place for approximately 10 s before slowly withdrawing it, and immediately apply gentle pressure to the injection site with a sterile cotton swab to absorb any leaked cell suspension. If leakage is observed, thoroughly rinse the injection site with sterile distilled water to remove and lyse free-floating tumor cells. Hypotonic distilled water is recommended for this step, as isotonic solutions do not lyse tumor cells and are therefore less effective in reducing the risk of peritoneal tumor seeding.

## Resource availability

### Lead contact

Further information and requests for resources and reagents should be directed to the lead contact, Lukas F. Mager (lukas.mager@med.uni-tuebingen.de).

### Technical contact

Technical questions on executing this protocol should be directed to and will be answered by the technical contact, Thi Trang Le (trang.le-thi@med.uni-tuebingen.de).

### Materials availability

This protocol did not generate any unique reagents.

### Data and code availability

This protocol did not generate any code or large datasets.

## Acknowledgments

L.F.M., T.T.L., and this project are funded through the 10.13039/501100005972German Cancer Aid (Max-Eder program). M.-L.P. is funded by 10.13039/100009139DZIF (Clinical-leave stipend).

We thank the staff of the M3 germ-free facility (M3GF) for their support with gnotobiotic animal handling and sterile workflow procedures. We also thank Dr. Jan Homolak, Isa-Maria Klink, and Dr. Umesh Gautam for their additional assistance.

We acknowledge support by Open Access Publishing Fund of 10.13039/501100002345University of Tübingen.

## Author contributions

Conceptualization, L.F.M. and R.B.; data acquisition and analysis T.T.L. and M.-L.P.; writing and manuscript revision, T.L., M.-L.P., C.K.S.-T., R.B., and L.F.M.; funding acquisition, L.F.M.

## Declaration of interests

The authors declare no competing interests.
